# Low Level of Colistin Resistance and *mcr* Genes Presence in *Salmonella* spp.: Evaluation of Isolates Collected between 2000 and 2020 from Animals and Environment

**DOI:** 10.3390/antibiotics11020272

**Published:** 2022-02-19

**Authors:** Fabrizio Bertelloni, Giulia Cagnoli, Barbara Turchi, Valentina Virginia Ebani

**Affiliations:** Department of Veterinary Science, University of Pisa, 56124 Pisa, Italy; g.cagnoli@studenti.unipi.it (G.C.); barbara.turchi@unipi.it (B.T.); valentina.virginia.ebani@unipi.it (V.V.E.)

**Keywords:** *Salmonella*, colistin resistance, *mcr*, animals

## Abstract

Salmonellosis is one of the most important zoonoses in Europe and the world. Human infection may evolve in severe clinical diseases, with the need for hospitalization and antimicrobial treatment. Colistin is now considered an important antimicrobial to treat infections from multidrug- resistant Gram-negative bacteria, but the spreading of mobile colistin-resistance (*mcr*) genes has limited this option. We aimed to evaluate colistin minimum inhibitory concentration and the presence of *mcr* (*mcr-1* to *mcr-9*) genes in 236 *Salmonella* isolates previously collected from different animals and the environment between 2000 and 2020. Overall, 17.79% of isolates were resistant to colistin; no differences were observed in relation to years of isolation (2000–2005, 2009–2014, and 2015–2020), *Salmonella enterica* subspecies (*enterica*, *salamae*, *diarizonae*, and *houtenae*), origin of samples (domestic animals, wildlife, and environment), or animal category (birds, mammals, and reptiles); only recently isolated strains from houseflies showed the most resistance. Few isolates (5.93%) scored positive for *mcr* genes, in particular for *mcr-1*, *mcr-2*, *mcr-4*, *mcr-6*, and *mcr-8*; furthermore, only 2.54% of isolates were *mcr*-positive and colistin-resistant. Detected resistance to colistin was equally distributed among all examined *Salmonella* isolates and not always related to the presence of *mcr* genes.

## 1. Introduction

Colistin is an old antibiotic, discovered in 1947, and belonging to the class of polymyxins. The target of this antimicrobial is the outer membrane lipopolysaccharides (LPS) of Gram-negative bacteria [1]. In human medicine, colistin use has been largely abandoned since the mid-1970s as a consequence of some adverse events, primarily nephrotoxicity and neurotoxicity, combined with the discovery of new antibiotics [2]. In contrast, colistin was largely used in veterinary medicine worldwide to treat infections, mainly from Enterobacteriaceae, in terrestrial and aquatic animals. Furthermore, it was used for decades as a growth promoter in farm animals in many countries; this practice is still allowed in some territories [3]. The increase in antimicrobial resistance led, by the mid-1990s, to the rediscovery of colistin for human therapy as a last-resort antibiotic in multidrug-resistant Gram-negative infections [2,4]. Consequently, policies for the reduction in use of colistin in animals were advanced in many countries, although the resistance to this antimicrobial was not so diffuse [1,5]. Resistance to colistin remained a rare event for years, probably because it is encoded chromosomally and consequently transferred only vertically [2,5].

In 2015, the first plasmid gene able to confer resistance to colistin, *mcr-1*, was detected in *Escherichia coli* in China [6]. Subsequently, different variants of this gene, from *mcr-2* to *mcr-10*, and numerous subvariants have been reported [7,8]. Surprisingly, retrospective investigations highlighted the presence of *mcr* genes in strains isolated from the 1980s, but particularly in bacteria cultured in the last twenty years [9,10,11]. However, before 2015, colistin resistance was not considered a serious threat and resistance levels were generally low [12]. Starting in 2015, reports of colistin-resistant and *mcr*-positive bacteria increased; now, these events are reported worldwide in many Gram-negative bacteria [12,13].

Despite *E. coli* remaining the main bacterial species involved in this phenomenon, colistin resistance and *mcr*-mediated resistance were also reported in *Salmonella* [14]. Phenotypic resistance to colistin in *Salmonella* is variable in relation to serotypes, year of isolation, source (humans, animals, food, and environment), and geographic area. However, percentages of resistant strains are generally low, ranging between about 1% and 12% [15,16,17,18], with some exceptions (40%) [19]. The gene *mcr-1* was the most frequently detected in *Salmonella*, but *mcr-2*, *mcr-3*, *mcr-4*, *mcr-5*, and *mcr-9* were also detected [20]. Domestic animals were identified as the main reservoir of *mcr*-positive salmonellae and *S.* ser Typhimurium as the most frequent serovar carrying these genes [14,20]. However, detection rate of *mcr*-positive *Salmonella* strains is generally low [17,18,21], with some exceptions [22]. As reported for other bacteria, the direct relation between the presence of *mcr* genes and colistin resistance in *Salmonella* has not been fully clarified; these genes have also been found in susceptible strains [15], and, frequently, only a small number of resistant strains carry these genes [16,17,18], suggesting other mechanisms of resistance [2]. In *S. enterica*, different chromosomally encoded mechanisms were identified as conferring resistance to colistin. In particular, the addition of 4-amino-4-deoxy-L-arabinose (L-Ara4N) and phosphoethanolamine (PEtN) to the phosphate groups of lipid A causes the alteration of the LPS structure; this event alters the primary target of colistin. The *arnBCADTEF* and *pmrCAB* operons are responsible for this process; they are up-regulated by the two-component regulatory systems (TCSs) PmrA/PmrB and PhoP/PhoQ. Overactivation of TCSs by external stimuli or by mutations may lead to the hyper-expression of *arnBCADTEF* and *pmrCAB*. Mutations in *mgrB*, a suppressor gene of the PhoP/PhoQ system, may produce the same result. Deacylation of lipid A, mediated by *pagL*, *rpoN*, and *ipxM* genes, may be another mechanism mediating colistin resistance, although less common in *S. enterica* than in other bacteria, such as *Acinetobacter baumannii*. Finally, it has been proposed that alteration of efflux pumps (*sapABCDF* operon) and porins by cytoplasmic molecules (*ydeI*/*ompD* system) can increase bacterial resistance to polymyxins [2,13,23,24].

The aim of this study was to investigate the colistin resistance and presence of *mcr* genes (*mcr-1* to *mcr-9*) in *Salmonella* strains isolated from animals or related environments, including strains cultured from atypical samples (reptiles and wild animals) and belonging to serotypes that are not frequently detected or investigated, collected between 2000 and 2020.

## 2. Results

### 2.1. Evaluation of Minimum Inhibitory Concentration (MIC) for Colistin

Overall, 42/236 (17.79%) isolates showed resistance to colistin; most of them, 33/236 (13.98%), showed an MIC equal to the breakpoint, 4 µg/mL. Consequently, only nine (3.81%) strains had a higher MIC. In particular, one (0.42%) isolate had an MIC of 8 µg/mL, one (0.42%) isolate of 16 µg/mL, one (0.42%) isolate of 256 µg/mL and six (2.54%) isolates had an MIC higher than 256 µg/mL.

Considering the years of isolation of the investigated strains and the first report of *mcr* genes in 2015, to evaluate the distribution over time of colistin resistance, isolates were arranged in three groups: salmonellae collected between 2000 and 2005, between 2009 and 2014, and finally, between 2015 and 2020 (no isolates were collected between 2006 and 2008). Percentages of resistant strains collected between 2000–2005 and 2009–2014 were very similar, 15.17% (17/112) and 15.27% (11/72), respectively, and no statistical differences emerged (*p* > 0.05). Although the percentage of resistant salmonellae isolated in the period 2015–2020 was higher, 28.00% (14/50), this difference was not statistically significant (*p* > 0.05). Table 1 shows detailed data on distribution of MICs over time in examined *Salmonella* strains.

Considering the source of the strains, 174 isolates were from domestic animals, including pets, 51 isolates were from wild animals, including houseflies and crayfish, and 11 isolates were from environmental and feed samples. In detail, 13 isolates were cultured from Arthropoda (housefly and crayfish), 51 from birds, 63 from mammals, and 98 from reptiles. Of strains from domestic animals, 16.67% (29/174) showed resistance to colistin, whereas 23.53% (12/51) and 9.09% (1/11) of isolates from wild animals and environmental samples were resistant, respectively (Table 2); no statistical differences were observed (*p* > 0.05). Likewise, 38.46% (5/13), 21.57% (11/51), 22.22% (14/63), and 11.22% (11/98) of *Salmonella* isolates from Arthropoda, birds, mammals, and reptiles showed resistance to colistin, respectively (Table 3); no statistical differences emerged among strains from birds, mammals, and reptiles (*p* > 0.05), whereas isolates from Arthropoda appeared more resistant than salmonellae from other animals (*p* < 0.05).

All analyzed strains belonged to the species *S. enterica*. In particular, 202 isolates belonged to subspecies *enterica*, 7 to subspecies *salamae*, 9 to subspecies *diarizonae*, and 14 to subspecies *houtenae*; finally, 4 strains were in the R phase. Table 4 shows the distribution of MIC values in relation to *Salmonella* subspecies. Considering the MIC results, 16.83% (34/202) of *S. enterica* sub. *enterica* isolates showed resistance, 14.29% (1/7) of *S. enterica* sub. *salamae* isolates, 22.22% (2/9) of *S. enterica* sub. *diarizonae* isolates, and 21.43% (3/14) of *S. enterica* sub. *houtenae* isolates showed resistance to colistin; 50.00% of R-phase strains were resistant. No statistical differences emerged among the different subspecies (*p* > 0.05).

A serovar-to-serovar comparison was not feasible because, for most serovars, only one isolate was available. We compared the data relating to the six serovars most frequently detected in human infections in EU between 2015 and 2019: Enteritidis, Typhimurium, Typhimurium monophasic variants, Infantis, Derby, and Newport [25]. As shown in Table 5, 40.00% of *S.* ser. Enteritidis, 20.69% (6/29) of *S.* ser. Typhimurium, 14.9% (1/7) of *S.* ser. Typhimurium monophasic variants, 25.00% (2/8) of *S.* ser. Infantis, 12.50% (2/16) of *S.* ser. Derby, and 0.00% (0/3) of *S.* ser. Newport showed resistance to colistin; no statistical differences were observed (*p* > 0.05).

### 2.2. Molecular Detection of mcr Genes

None of the isolates were positive for *mcr-3*, *mcr-5*, *mcr-7*, or *mcr-9* genes. The most detected gene was *mcr-2*, with 7/236 (2.96%) positive isolates, followed by *mcr-4*, with 4/236 (1.69%) positive isolates, and *mcr-1* and *mcr-8*, with 2/236 (0.84%) positive isolates each; only 1/236 (0.42%) scored positive for *mcr-6*. Overall, 14/236 (5.93%) isolates were positive for *mcr* genes; only two isolates scored positive for more than one gene. Six isolates were collected in 2019 from different specimens and showed phenotypic resistance to colistin. Eight *Salmonella* strains, collected in 2002 from reptiles, showed susceptibility to colistin, although they were positive for the investigated resistance genes. Table 6 shows detailed information about *mcr*-positive strains.

## 3. Discussion

*Salmonella* is one of the most important food-borne zoonotic pathogens worldwide. In Europe, salmonellosis is the second most prevalent zoonosis considering the total number of confirmed cases and the most prevalent considering the total number of outbreaks. In 2019, in EU, *Salmonella* was responsible for 87,923 confirmed human cases, 16,628 hospitalizations, and 140 deaths [25]. In Europe, antimicrobial resistance, mainly multidrug resistance, was generally lower in *Salmonella* than in other Enterobacteriaceae, with differences related to serotype and geographic territory [26]. Colistin was recently reintroduced in human therapy and it is now considered a critically important antimicrobial [27]. For this reason, the discovery of mobile genes conferring colistin resistance caused many concerns. These genes were more commonly observed among *E. coli* strains, but they were also detected in other Gram-negative bacteria, such as *Salmonella* [28].

The number of studies on phenotypic colistin resistance in *Salmonella* is limited. Past works were usually based on the disk diffusion test, a method not suitable for colistin resistance detection [1]; on the other hand, recent investigations usually focused on *mcr*-positive strains. In our study, the percentage of colistin-resistant strains was 17.79%, which is slightly higher than the data reported in recent literature. Recent surveys registered percentages of colistin-resistant *Salmonella* strains ranging between 1% and 12% [15,17,18,29,30]. These differences may be due to many factors: source, geographic area, and serotype. It is interesting to note that no differences were observed in relation to years of isolation, suggesting a basal level of diffused resistance. Unfortunately, our study suffers from the heterogeneity of samples from which *Salmonella* isolates were cultured; thus, it was not possible to draw a conclusion in terms of monitoring over time. However, this limitation may also represent a strength of the study, showing a condition largely spread among the *Salmonella* genus and not restricted to strains coming from a particular matrix. To underline this hypothesis, no differences were observed among strains from wild animals, domestic animals, or environment, or from different animal categories: birds, mammals, and reptiles. *Salmonella* isolates from houseflies (*Musca domestica*) were statistically the most resistant strains. Considering the behavior of houseflies, the obtained results may suggest that these insects might be involved in the diffusion of colistin-resistant salmonellae.

Most of the available studies in the literature focused on a subset of serovars, the ones mainly involved in human and animal salmonellosis, such as Typhimurium and Enteritidis [14,16,20]. Few data are available on colistin resistance in *Salmonella* belonging to uncommon serovars or subspecies [31]. In our investigation, more than 70 different serovars, belonging to four subspecies, were included. In relation to phenotypic colistin resistance, no statistical differences were observed among the subspecies. Furthermore, no statistical differences emerged among the six serovars most frequently detected in human infections in EU between 2015 and 2019 [25]. These findings support the hypothesis of a diffused resistance among tested salmonellae. Moreover, our results show that the less investigated serovars may also be involved in the phenomenon of colistin resistance.

Regarding mobile colistin-resistance genes, few isolates scored positive (5.93%) for one or more genes. This finding is in agreement with other studies reporting a percentage of *mcr*-positive *Salmonella* strains ranging between 0.6% and 3.3% [15,17,18,19,29]. The slightly higher positivity detected in our survey may be related to the extended set of investigated genes. Our data confirm the low circulation of these genes in the *Salmonella* genus compared to other Enterobacteriaceae, especially *E. coli. E. coli* seems to be the primary species having *mcr* genes [9,28,32], also confirmed by the high prevalence of *mcr*-positive *E. coli* strains (44.6%) isolated from wild boar from the same geographic area [33].

The low detection rate of *mcr* genes compared to found phenotypic resistance suggests the low involvement of these genes in colistin resistance in *Salmonella*, as also supposed by other authors [17,29]. Detected resistance may be related to other chromosome-related mechanisms. From this point of view, the TCSs (*pmrA*/*pmrB* and *phoP*/*phoQ*) play a key role in colistin resistance in *Salmonella*. Mutations occurring in these genes or in their regulatory genes, such as *mgrB* for PhoP/Q, lead to the modification of lipid A and, consequently, the alteration of the colistin target [2,13,24]. Up-regulation of TCSs may also be the consequence of environmental stimuli, including low Mg^2+^ concentrations [2]. For this reason, cation-adjusted Mueller–Hinton medium was employed in MIC tests, as recommended by CLSI [34]. Other resistance mechanisms reported in *Salmonella* are related to efflux pumps and porins, but they seem to play a secondary role in colistin resistance in these bacteria [1].

Only 6/14 *mcr*-positive isolates were resistant to colistin. All *mcr*-positive and colistin-susceptible strains were from reptiles sampled in 2002. The gene *mcr*-1 was detected for the first time in *E. coli* in 2015 [6]. However, studies performed since 2015 have also shown the presence of this gene in previously obtained isolates [9], including *Salmonella* strains [14,20]. Currently, the exact relation between the presence of *mcr* genes and phenotypic resistance to colistin is controversial and far from being fully understood. In most studies, only colistin-resistant strains were screened for *mcr* and this aspect may cause a lack of information. Recent investigations also showed the abundant presence of these genes in susceptible bacteria [35]; furthermore, some *mcr* variants are unable to confer colistin resistance [31]. The obtained results highlighted the importance of carrying out both phenotypic and genotypic analyses to investigate the antimicrobial resistance.

The present research, which focused on the resistance to colistin in *Salmonella* strains cultured from different sources, suffers from some limitations. It would be useful to investigate if colistin-resistant and *mcr*-positive strains are also resistant to other antibiotics and whether they harbor other resistant genes, particularly at the plasmid level. Furthermore, we investigated only the involvement of *mcr* genes in colistin resistance. As reported above, other chromosomally related mechanisms induce resistance to colistin; the study of these systems may explain some resistances that were phenotypically detected.

## 4. Materials and Methods

### 4.1. Salmonella Strains Included in the Study

This investigation was conducted on 236 *Salmonella* spp. strains preserved in the biobank of the Laboratory of Infectious Disease of the Department of Veterinary Sciences, University of Pisa, Italy. All strains were collected during routine investigations or specific research. Most of the isolates came from apparently healthy animals. The strains were selected based on their pulsotype profile and/or taking into consideration epidemiological information. All strains were isolated from feces or organs of various domestic or wild animals (mammals, birds, reptiles, and arthropods), with the exception of 11 strains coming from environmental or feed samples collected at animal farms. Supplementary Table S1 shows information for each strain related to the type of sample, isolation year, species, subspecies, and serotype.

### 4.2. Evaluation of Minimum Inhibitory Concentration for Colistin

The broth microdilution method was employed for evaluation of minimum inhibitory concentrations (MICs) of colistin. The protocol described by CLSI was adopted [34]; cation-adjusted Mueller–Hinton (MH) broth (Oxoid, Milan, Italy) was used and colistin sulfate (CARLO ERBA Reagents, Cornaredo, Italy) was diluted 2-fold from 256 to 0.5 µg/mL. Isolates showing an MIC > 2 μg/mL were recorded as resistant [36].

### 4.3. Molecular Detection of mcr Genes

DNA was extracted from overnight agar cultures with a commercial kit, DNA Plus Kits (Zymo Research, Irvine, CA, USA), following the manufacturer’s guidelines.

Two multiplex PCR assays were employed: the first for the detection of genes *mcr-1*, *mcr-2*, *mcr-3*, *mcr-4*, and *mcr-5*; and the second to detect *mcr-6*, *mcr-7*, *mcr-8*, and *mcr-9* genes. The assays were executed using primers and protocols published elsewhere [37,38] and are shown in Table 7. PCR reactions were carried out in a volume of 50 µL including 25 µL EconoTaq PLUS GREEN 2X Master Mixes (Lucigen Corporation, Middleton, WI, USA), 0.5 µL of each primer (10 µM concentration), 3 µL of template DNA, and PCR-grade water to reach the final volume.

### 4.4. Statistical Analyses

Chi-square (X^2^) and Fisher (F) tests were used to evaluate the obtained data. In particular, statistical tests were used to compare distribution of resistant strains and positivity to *mcr* genes in relation to: year of isolation, source of isolates, *Salmonella* subspecies, and serovars. The statistical significance threshold was set at a *p*-value ≤ 0.05.

## 5. Conclusions

In the present study, we focused on the resistance to colistin in *Salmonella* strains isolated and collected over the last twenty years. No relevant differences emerged among the investigated isolates, suggesting a broad resistance in strains from different sources belonging to different serovars and isolated in different years. Phenotypic resistance was not always associated with *mcr* genes, confirming that this rising issue marginally involves *Salmonella* strains. The results of the resistance of the studied isolates to other antimicrobials have been reported in previous surveys. However, the present investigation may be improved by further data about other possible genetic mechanisms responsible for colistin resistance, as suggested by some authors who assumed that chromosomally related mechanisms are involved [2,13,24]. Programs of antibiotic-resistance monitoring should include *Salmonella* of uncommon serovars and isolated from atypical hosts to obtain more real and valuable information.

## Figures and Tables

**Table 1:** Distribution of the tested *Salmonella* strains in relation to minimum inhibitory concentration (MIC) values and years of isolation.

Years of Isolation	MIC Values (µg/mL)											Total
	≤0.5	1	2	4	8	16	32	64	128	256	>256	
2000–2005	30	26	39	14	0	1	0	0	0	1	1	112
2009–2014	16	26	19	10	1	0	0	0	0	0	0	72
2015–2020	12	8	16	9	0	0	0	0	0	0	5	50
Total	58	61	75	33	1	1	0	0	0	1	6	58

**Table 2:** Distribution of the tested *Salmonella* strains in relation to minimum inhibitory concentration (MIC) values and origin of samples.

	MIC Values (µg/mL)											Total
	≤0.5	1	2	4	8	16	32	64	128	256	>256	
Domestic animals	43	48	54	22	1	1	0	0	0	1	4	174
Wild animals	14	10	15	10	0	0	0	0	0	0	2	51
Environment	1	3	6	1	0	0	0	0	0	0	0	11
Total	58	61	75	33	1	1	0	0	0	1	6	236

**Table 3:** Distribution of the tested *Salmonella* strains in relation to minimum inhibitory concentration (MIC) values and animal categories.

	MIC Values (µg/mL)											Total
	≤0.5	1	2	4	8	16	32	64	128	256	>256	
Arthropoda	3	1	4	5	0	0	0	0	0	0	0	13
Birds	10	16	14	10	1	0	0	0	0	0	0	51
Mammals	14	15	20	8	0	0	0	0	0	0	6	63
Reptiles	30	26	31	9	0	1	0	0	0	1	0	98
Environment/ Feed	1	3	6	1	0	0	0	0	0	0	0	11
Total	58	61	75	33	1	1	0	0	0	1	6	236

**Table 4:** Distribution of the tested *Salmonella* strains in relation to minimum inhibitory concentration (MIC) values and subspecies.

Subspecies	MIC Values (µg/mL)											Total
	≤0.5	1	2	4	8	16	32	64	128	256	>256	
*enterica*	48	59	61	30	1	1	0	0	0	0	2	202
*salamae*	3	0	3	1	0	0	0	0	0	0	0	7
*diarizonae*	3	1	3	0	0	0	0	0	0	0	2	9
*houtenae*	3	1	7	0	0	0	0	0	0	1	2	14
R phase	1	0	1	2	0	0	0	0	0	0	0	4
Total	58	61	75	33	1	1	0	0	0	1	6	236

**Table 5:** Minimum inhibitory concentration (MIC) values obtained for *Salmonella* serovars most frequently detected in human infections in EU between 2015 and 2019.

Serovar	MIC Values (µg/mL)											Total
	≤0.5	1	2	4	8	16	32	64	128	256	>256	
Derby	4	8	2	1	0	0	0	0	0	0	1	16
Enteritidis	0	3	3	3	1	0	0	0	0	0	0	10
Infantis	1	2	3	2	0	0	0	0	0	0	0	8
Newport	1	2	0	0	0	0	0	0	0	0	0	3
TMV	0	5	1	1	0	0	0	0	0	0	0	7
Typhimurium	9	5	9	5	0	0	0	0	0	0	1	29

Legend: TMV = Typhimurium monophasic variant.

**Table 6:** Detailed information about the *mcr*-positive *Salmonella* isolates.

Isolate	Subspecies	Serovar	Animal	Sample	Year	MIC (µg/mL)	*mcr-1*	*mcr-2*	*mcr-4*	*mcr-6*	*mcr-8*
S356	*houtenae*	1,40:z_4_,z_23_:-	Donkey	Organs	2019	>256	-	+	-	-	-
S358	*houtenae*	1,40:z_4_,z_23_:-	Sheep	Organs	2019	>256	-	+	-	-	-
S374	*enterica*	Napoli	Housefly		2019	4	-	+	+	-	-
S375		R phase	Housefly		2019	4	-	-	+	-	-
S378		R phase	Housefly		2019	4	-	-	+	-	-
S386	*diarizonae*	50:r:1,5,7	Wild boar	Feces	2019	>256	+	-	-	-	-
R43	*enterica*	Trimndon	Reptile	Feces	2002	≤0.5	-	-	-	+	-
R108	*enterica*	Memphis	Reptile	Feces	2002	≤0.5	-	+	-	-	-
R164	*houtenae*	44:z_4_,z_23_:-	Reptile	Feces	2002	2	-	-	-	-	+
R161	*houtenae*	44:z_4_,z_23_:-	Reptile	Feces	2002	2	-	+	-	-	-
R173	*enterica*	Senftenberg	Reptile	Feces	2002	≤0.5	-	-	-	-	+
R300	*diarizonae*	50:z:z_52_	Reptile	Feces	2002	1	+	-	+	-	-
R112	*houtenae*	18:z_36_,z_23_:-	Reptile	Feces	2002	≤0.5	-	+	-	-	-
R126	*diarizonae*	48:z_4_,z_23_:-	Reptile	Feces	2002	2	-	+	-	-	-
Total							2	7	4	1	2

**Table 7:** Primers and protocols employed in PCR assays.

	Target Gene	Primer Name	Sequence (5′-3′)	Expected Size (bp)	Protocols	References
Multiplex 1	*mcr-1*	mcr1_320bp_fw	AGTCCGTTTGTTCTTGTGGC	320	Initial denaturation at 95 °C for 10 min, 25 cycles: denaturation at 95 °C for 30 s, annealing at 58 °C for 90 s, elongation at 72 °C for 60 s, Final elongation at 72 °C for 10 min.	[38]
		mcr1_320bp_rev	AGATCCTTGGTCTCGGCTTG			
	*mcr-2*	mcr2_700bp_fw	CAAGTGTGTTGGTCGCAGTT	715		
		mcr2_700bp_rev	TCTAGCCCGACAAGCATACC			
	*mcr-3*	mcr3_900bp_fw	AAATAAAAATTGTTCCGCTTATG	929		
		mcr3_900bp_rev	AATGGAGATCCCCGTTTTT			
	*mcr-4*	mcr4_1100bp_fw	TCACTTTCATCACTGCGTTG	1116		
		mcr4_1100bp_rev	TTGGTCCATGACTACCAATG			
	*mcr-5*	MCR5_fw	ATGCGGTTGTCTGCATTTATC	1644		
		MCR5_rev	TCATTGTGGTTGTCCTTTTCTG			
Multiplex 2	*mcr-6*	mcr-6_mp_fw	AGCTATGTCAATCCCGTGAT	252	Initial denaturation at 95 °C for 10 min, 30 cycles: denaturation at 95 °C for 30 s, annealing at 55 °C for 30 s, elongation at 72 °C for 60 s, Final elongation step at 72 °C for 10 min.	[37]
		mcr-6_mp_rev	ATTGGCTAGGTTGTCAATC			
	*mcr-7*	mcr-7_mp_fw	GCCCTTCTTTTCGTTGTT	551		
		mcr-7_mp_rev	GGTTGGTCTCTTTCTCGT			
	*mcr-8*	mcr-8_mp_fw	TCAACAATTCTACAAAGCGTG	856		
		mcr-8_mp_rev	AATGCTGCGCGAATGAAG			
	*mcr-9*	mcr-9_mp_fw	TTCCCTTTGTTCTGGTTG	1011		
		mcr-9_mp_rev	GCAGGTAATAAGTCGGTC			

## Data Availability

The data presented in this study are available within the article and Supplementary Material.

## Supplementary Materials

The following are available online at https://www.mdpi.com/article/10.3390/antibiotics11020272/s1, Table S1: Detailed information about analyzed *Salmonella* isolates.
