# Supplementary material for: Low Level of Colistin Resistance and mcr Genes Presence in Salmonella spp.: Evaluation of Isolates Collected between 2000 and 2020 from Animals and Environment
Source: Antibiotics (Basel). 2022 Feb 19;11(2):272. doi: 10.3390/antibiotics11020272 (PMC8868313; doi:10.3390/antibiotics11020272)
Supplement: Supplementary file 1 [file antibiotics-11-00272-s001.zip › antibiotics-1558210-supplementary.pdf]

Supplementary Material

Low Level of Colistin Resistance and mcr Genes Presence in Salmonella spp.: Evaluation of Isolates Collected between 2000 and 2020 from Animals and Environment

Fabrizio Bertelloni *, Giulia Cagnoli, Barbara Turchi and Valentina Virginia Ebani

Department of Veterinary Science, University of Pisa, 56124 Pisa, Italy; g.cagnoli@studenti.unipi.it (G.C.); barbara.turchi@unipi.it (B.T.); valentina.virginia.ebani@unipi.it (V.V.E.)

* Correspondence: fabrizio.bertelloni@unipi.it

Table S1. Detailed information about analyzed *Salmonella* isolates.

Isolate N°	subspecies	serovar	Animal	Kind of Sample	Isolation Year	MIC (µg/mL)	*mcr* genes								
							mcr-1	***mcr-2***	***mcr-3***	***mcr-4***	***mcr-5***	***mcr-6***	***mcr-7***	***mcr-8***	***mcr-9***
S1	*enterica*	Mbandaka		F	2009	2	-	-	-	-	-	-	-	-	-
S3	*enterica*	Mbandaka		E	2009	1	-	-	-	-	-	-	-	-	-
S5	*enterica*	Agona		F	2010	2	-	-	-	-	-	-	-	-	-
S7	*enterica*	Montevideo		F	2010	2	-	-	-	-	-	-	-	-	-
S9	*enterica*	TMV	Poultry	O	2012	1	-	-	-	-	-	-	-	-	-
S10	*enterica*	Saintpaul	Poultry	O	2012	≤ 0.5	-	-	-	-	-	-	-	-	-
S11	*enterica*	Typhimurium	Swine	O	2010	4	-	-	-	-	-	-	-	-	-
S12	*enterica*	TMV	Poultry	O	2012	1	-	-	-	-	-	-	-	-	-
S13	*enterica*	Abony	Reptile	O	2010	1	-	-	-	-	-	-	-	-	-
S14	*enterica*	Richmond	Reptile	O	2010	1	-	-	-	-	-	-	-	-	-
S17	*enterica*	Richmond	Reptile	O	2010	1	-	-	-	-	-	-	-	-	-
S18	*enterica*	Richmond	Bat	O	2010	1	-	-	-	-	-	-	-	-	-
S19	*enterica*	Newport		E	2010	1	-	-	-	-	-	-	-	-	-
S22	*enterica*	London		E	2010	≤ 0.5	-	-	-	-	-	-	-	-	-
S24	*enterica*	London		E	2010	2	-	-	-	-	-	-	-	-	-
S28	*enterica*	Typhimurium	Poultry	O	2010	≤ 0.5	-	-	-	-	-	-	-	-	-
S29	*enterica*	Derby	Swine	O	2011	≤ 0.5	-	-	-	-	-	-	-	-	-
S31	*enterica*	Derby	Swine	O	2011	≤ 0.5	-	-	-	-	-	-	-	-	-
S33	*enterica*	Derby	Swine	O	2011	≤ 0.5	-	-	-	-	-	-	-	-	-

S34	*enterica*	Derby	Swine	O	2011	1	-	-	-	-	-	-	-	-	-
S39	*enterica*	Derby	Swine	O	2011	1	-	-	-	-	-	-	-	-	-
S41	*enterica*	Hermannswerder	Reptile	S	2004	1	-	-	-	-	-	-	-	-	-
S42	*enterica*	Typhimurium	Pigeon	S	2003	4	-	-	-	-	-	-	-	-	-
S57	*enterica*	Abaetetuba	Reptile	S	2004	2	-	-	-	-	-	-	-	-	-
S58	*enterica*	Nyanza	Reptile	S	2004	1	-	-	-	-	-	-	-	-	-
S59	*enterica*	Ebrie	Reptile	S	2004	2	-	-	-	-	-	-	-	-	-
S60	*enterica*	Ebrie	Reptile	S	2004	4	-	-	-	-	-	-	-	-	-
S61	*enterica*	50:b:z6	Reptile	S	2004	2	-	-	-	-	-	-	-	-	-
S62	*enterica*	Ebrie	Reptile	S	2004	1	-	-	-	-	-	-	-	-	-
S63	*enterica*	1,4,5,12:-:-	Reptile	S	2004	2	-	-	-	-	-	-	-	-	-
S64	*salamae (II)*	9,12:z_{29}:1,5	Reptile	S	2004	4	-	-	-	-	-	-	-	-	-
S65	*enterica*	Miami	Reptile	S	2004	4	-	-	-	-	-	-	-	-	-
S66	*enterica*	Miami	Reptile	S	2004	1	-	-	-	-	-	-	-	-	-
S67	*enterica*	Miami	Reptile	S	2004	1	-	-	-	-	-	-	-	-	-
S68	*enterica*	Tornow	Reptile	S	2004	2	-	-	-	-	-	-	-	-	-
S69	*enterica*	1,4,5,12:-:-	Reptile	S	2004	2	-	-	-	-	-	-	-	-	-
S70	*enterica*	1,4,5,12:-:-	Reptile	S	2004	1	-	-	-	-	-	-	-	-	-
S71	*enterica*	Poona	Reptile	S	2004	≤ 0.5	-	-	-	-	-	-	-	-	-
S72	*enterica*	Goeteborg	Reptile	S	2004	2	-	-	-	-	-	-	-	-	-
S73	*enterica*	Tornow	Reptile	S	2004	2	-	-	-	-	-	-	-	-	-
S74	*enterica*	Hermannswerder	Reptile	S	2004	2	-	-	-	-	-	-	-	-	-
S75	*enterica*	Montevideo		E	2002	2	-	-	-	-	-	-	-	-	-
S76	*enterica*	Derby	Swine	O	2000	2	-	-	-	-	-	-	-	-	-
S77	*enterica*	Mbandaka	Swine	O	2000	2	-	-	-	-	-	-	-	-	-
S78	*enterica*	Typhimurium	Swine	O	2000	2	-	-	-	-	-	-	-	-	-
S79	*enterica*	Give	Swine	O	2000	4	-	-	-	-	-	-	-	-	-
S80	*enterica*	Livingston	Swine	O	2000	1	-	-	-	-	-	-	-	-	-
S82	*enterica*	Infantis	Swine	O	2000	4	-	-	-	-	-	-	-	-	-
S83	*enterica*	Typhimurium	Swine	O	2000	> 256	-	-	-	-	-	-	-	-	-
S84	*enterica*	Pomona	Reptile	S	2004	4	-	-	-	-	-	-	-	-	-
S85	*enterica*	Tiergarten	Reptile	S	2004	4	-	-	-	-	-	-	-	-	-
S86	*enterica*	Tiergarten	Reptile	S	2004	4	-	-	-	-	-	-	-	-	-

S87	*enterica*	Kumasi	Reptile	S	2004	16	-	-	-	-	-	-	-	-	-
S88	*enterica*	Pomona	Reptile	S	2004	2	-	-	-	-	-	-	-	-	-
S89	*enterica*	Miami	Reptile	S	2004	4	-	-	-	-	-	-	-	-	-
S90	*enterica*	Miami	Reptile	S	2004	2	-	-	-	-	-	-	-	-	-
S91	*enterica*	Agona		E	2004	1	-	-	-	-	-	-	-	-	-
S92	*enterica*	Agona		E	2004	2	-	-	-	-	-	-	-	-	-
S93	*enterica*	Infantis		E	2004	4	-	-	-	-	-	-	-	-	-
S94	*enterica*	Choleraesuis	Swine†	O	2005	1	-	-	-	-	-	-	-	-	-
S100	*enterica*	Typhimurium	Swine	O	2005	2	-	-	-	-	-	-	-	-	-
S101	*enterica*	1,4,5,12:-:-	Reptile	S	2004	1	-	-	-	-	-	-	-	-	-
S102	*enterica*	1,4,5,12:-:-	Reptile	S	2004	2	-	-	-	-	-	-	-	-	-
S103	*enterica*	Typhimurium	Pigeon	S	2003	2	-	-	-	-	-	-	-	-	-
S106	*enterica*	Typhimurium	Pigeon	S	2003	4	-	-	-	-	-	-	-	-	-
S108	*enterica*	Typhimurium	Reptile	S	2004	2	-	-	-	-	-	-	-	-	-
S109	*enterica*	Typhimurium	Poultry	O	2012	2	-	-	-	-	-	-	-	-	-
S110	*enterica*	Livingston	Dog†	S	2003	2	-	-	-	-	-	-	-	-	-
S111	*enterica*	Thompson	Poultry	O	2011	2	-	-	-	-	-	-	-	-	-
S149	*enterica*	Typhimurium	Pigeon	O	2012	2	-	-	-	-	-	-	-	-	-
S150	*enterica*	Typhimurium	Pigeon	O	2012	2	-	-	-	-	-	-	-	-	-
S154	*enterica*	Derby	Parrot	S	2012	1	-	-	-	-	-	-	-	-	-
S157	*enterica*	Derby	Parrot	S	2012	1	-	-	-	-	-	-	-	-	-
S161	*enterica*	Newport	Poultry	O	2012	≤ 0.5	-	-	-	-	-	-	-	-	-
S163	*enterica*	Blockley	Poultry	O	2012	4	-	-	-	-	-	-	-	-	-
S165	*enterica*	Bredeney	Poultry	O	2012	4	-	-	-	-	-	-	-	-	-
S167	*enterica*	Bredeney	Poultry	O	2012	2	-	-	-	-	-	-	-	-	-
S168	*enterica*	Blockley	Poultry	O	2012	2	-	-	-	-	-	-	-	-	-
S169	*enterica*	Kapemba	Swine	O	2012	2	-	-	-	-	-	-	-	-	-
S171	*enterica*	Bredeney	Raptor	S	2013	1	-	-	-	-	-	-	-	-	-
S173	*enterica*	Derby	Swine	O	2013	4	-	-	-	-	-	-	-	-	-
S174	*enterica*	Montevideo	Reptile	S	2002	≤ 0.5	-	-	-	-	-	-	-	-	-
S176	*enterica*	Typhimurium	Poultry	S	2013	2	-	-	-	-	-	-	-	-	-
S177	*enterica*	Infantis	Swine	S	2013	2	-	-	-	-	-	-	-	-	-
S178	*enterica*	Infantis	Raptor	S	2014	1	-	-	-	-	-	-	-	-	-

S179	*enterica*	Derby	Swine	S	2014	1	-	-	-	-	-	-	-	-	-
S184	*enterica*	Kapemba	Swine	S	2014	2	-	-	-	-	-	-	-	-	-
S190	*enterica*	Typhimurium	Pigeon	O	2014	1	-	-	-	-	-	-	-	-	-
S192	*enterica*	Infantis	Swine	S	2014	1	-	-	-	-	-	-	-	-	-
S193	*enterica*	Derby	Swine	S	2014	1	-	-	-	-	-	-	-	-	-
S194	*enterica*	Agona	Reptile	S	2002	2	-	-	-	-	-	-	-	-	-
S195	*enterica*	Derby	Swine	S	2014	2	-	-	-	-	-	-	-	-	-
S203	*enterica*	Group O:4	Swine	S	2014	≤ 0.5	-	-	-	-	-	-	-	-	-
S207	*enterica*	Infantis	Poultry	S	2014	2	-	-	-	-	-	-	-	-	-
S215	*enterica*	Coeln	Wild Bird	O	2014	4	-	-	-	-	-	-	-	-	-
S216	*enterica*	Napoli	Wild Boar	O	2015	4	-	-	-	-	-	-	-	-	-
S217	*enterica*	Enteritidis	Poultry	S	2013	4	-	-	-	-	-	-	-	-	-
S218	*enterica*	Enteritidis	Poultry	O	2013	4	-	-	-	-	-	-	-	-	-
S219	*enterica*	Enteritidis	Poultry	O	2013	4	-	-	-	-	-	-	-	-	-
S220	*enterica*	Enteritidis	Poultry	O	2013	2	-	-	-	-	-	-	-	-	-
S221	*enterica*	Enteritidis	Poultry	O	2013	2	-	-	-	-	-	-	-	-	-
S230	*enterica*	Enteritidis	Hedgehog	O	2014	1	-	-	-	-	-	-	-	-	-
S232	*enterica*	Enteritidis	Poultry	O	2014	2	-	-	-	-	-	-	-	-	-
S233	*enterica*	Enteritidis	Poultry	O	2013	8	-	-	-	-	-	-	-	-	-
S234	*enterica*	Enteritidis	Poultry	S	2012	1	-	-	-	-	-	-	-	-	-
S236	*enterica*	Enteritidis	Poultry	S	2011	1	-	-	-	-	-	-	-	-	-
S237	*enterica*	TMV	Poultry	O	2012	1	-	-	-	-	-	-	-	-	-
S238	*enterica*	TMV	Poultry	O	2012	1	-	-	-	-	-	-	-	-	-
S239	*enterica*	TMV	Poultry	S	2013	2	-	-	-	-	-	-	-	-	-
S240	*enterica*	Typhimurium	Poultry	S	2013	1	-	-	-	-	-	-	-	-	-
S241	*enterica*	Typhimurium	Poultry	S	2013	1	-	-	-	-	-	-	-	-	-
S244	*enterica*	TMV	Poultry	O	2013	1	-	-	-	-	-	-	-	-	-
S245	*enterica*	Typhimurium	Poultry	O	2013	≤ 0.5	-	-	-	-	-	-	-	-	-
S246	*enterica*	Typhimurium	Pigeon	O	2014	≤ 0.5	-	-	-	-	-	-	-	-	-
S247	*enterica*	Typhimurium	Quail	S	2014	≤ 0.5	-	-	-	-	-	-	-	-	-
S248	*enterica*	Typhimurium	Badger	O	2014	≤ 0.5	-	-	-	-	-	-	-	-	-
S250	*enterica*	Typhimurium	Poultry	O	2014	1	-	-	-	-	-	-	-	-	-
S251	*enterica*	Typhimurium	Poultry	O	2014	≤ 0.5	-	-	-	-	-	-	-	-	-

S252	*enterica*	Typhimurium	Poultry	O	2014	≤ 0.5	-	-	-	-	-	-	-	-	-
S253	*enterica*	Typhimurium	Sparrow	O	2014	≤ 0.5	-	-	-	-	-	-	-	-	-
S258	*enterica*	Typhimurium	Poultry	O	2014	4	-	-	-	-	-	-	-	-	-
S261	*enterica*	Typhimurium	Poultry	S	2014	4	-	-	-	-	-	-	-	-	-
S262	*enterica*	Derby	Swine	Carcass	2015	> 256	-	-	-	-	-	-	-	-	-
S269	*enterica*	Bredeney	Raptor	S	2015	2	-	-	-	-	-	-	-	-	-
S272	*enterica*	Derby	Swine	S	2016	≤ 0.5	-	-	-	-	-	-	-	-	-
S276	*enterica*	Rissen	Swine	S	2016	1	-	-	-	-	-	-	-	-	-
S293	*enterica*	Bovismorbificans	Swine	O	2016	2	-	-	-	-	-	-	-	-	-
S311	*enterica*	Paratyphi B	Swine	O	2016	2	-	-	-	-	-	-	-	-	-
S315	*enterica*	Derby	Swine	O	2016	1	-	-	-	-	-	-	-	-	-
S317	*enterica*	London	Swine	S	2016	4	-	-	-	-	-	-	-	-	-
S322	*enterica*	Typhimurium	Swine	S	2016	2	-	-	-	-	-	-	-	-	-
S323	*enterica*	Derby	Swine	O	2016	1	-	-	-	-	-	-	-	-	-
S324	*houtenae (IV)*	40:z_4,z_{23}:-	Swine	O	2016	2	-	-	-	-	-	-	-	-	-
S333	*enterica*	TMV	Swine	S	2017	4	-	-	-	-	-	-	-	-	-
S335	*enterica*	Typhimurium	Wild Bird	S	2018	1	-	-	-	-	-	-	-	-	-
S336	*enterica*	Manhattan	Crayfish	O	2018	2	-	-	-	-	-	-	-	-	-
S339	*enterica*	Veneziana	Red Panda	O	2018	2	-	-	-	-	-	-	-	-	-
S341	*enterica*	Napoli	Wolf	S	2018	1	-	-	-	-	-	-	-	-	-
S343	*enterica*	Napoli	Wild goat	S	2018	4	-	-	-	-	-	-	-	-	-
S344	*enterica*	Napoli	Fox	S	2018	2	-	-	-	-	-	-	-	-	-
S345	*enterica*	Newport	Wild Boar	O	2018	1	-	-	-	-	-	-	-	-	-
S348	*enterica*	Infantis	Wild Boar	O	2019	2	-	-	-	-	-	-	-	-	-
S350	*diarizonae (IIIb)*	50:r:1,5,7	Wild Boar	O	2019	> 256	-	-	-	-	-	-	-	-	-
S353	*salamae (II)*	42:z:1,5	Wild Boar	O	2019	≤ 0.5	-	-	-	-	-	-	-	-	-
S354	*diarizonae (IIIb)*	50:r:1,5,7	Wild Boar	O	2019	≤ 0.5	-	-	-	-	-	-	-	-	-
S356	*houtenae (IV)*	1,40:z_4,z_{23}:-	Donkey	O	2019	> 256	-	+	-	-	-	-	-	-	-
S358	*houtenae (IV)*	1,40:z_4,z_{23}:-	Sheep	O*	2019	> 256	-	+	-	-	-	-	-	-	-
S360	*enterica*	London	Housefly	O*	2019	2	-	-	-	-	-	-	-	-	-
S362	*enterica*	Rubislaw	Housefly	O*	2019	4	-	-	-	-	-	-	-	-	-
S365	*enterica*	Napoli	Housefly	O*	2019	1	-	-	-	-	-	-	-	-	-
S366	*enterica*	Rubislaw	Housefly	O*	2019	4	-	-	-	-	-	-	-	-	-

S369	*enterica*	Kisarawe	Housefly	O*	2019	≤ 0.5	-	-	-	-	-	-	-	-	-
S370	*enterica*	Kentucky	Housefly	O*	2019	≤ 0.5	-	-	-	-	-	-	-	-	-
S372	*enterica*	Kentucky	Housefly	O*	2019	≤ 0.5	-	-	-	-	-	-	-	-	-
S373		R fase	Housefly	O*	2019	2	-	-	-	-	-	-	-	-	-
S374	*enterica*	Napoli	Housefly	O*	2019	4	-	+	-	+	-	-	-	-	-
S375		R fase	Housefly	O*	2019	4	-	-	-	+	-	-	-	-	-
S377	*enterica*	Napoli	Housefly	O*	2019	2	-	-	-	-	-	-	-	-	-
S378		R fase	Housefly	O*	2019	4	-	-	-	+	-	-	-	-	-
S381	*diarizonae (IIIb)*	50:r:1,5,7	Wild Boar	O*	2019	2	-	-	-	-	-	-	-	-	-
S383		R fase	Wild Boar	S	2019	≤ 0.5	-	-	-	-	-	-	-	-	-
S385	*enterica*	Napoli	Wild Boar	S	2019	2	-	-	-	-	-	-	-	-	-
S386	*diarizonae (IIIb)*	50:r:1,5,7	Wild Boar	S	2019	> 256	+	-	-	-	-	-	-	-	-
S387	*enterica*	Rubislaw	Wild Boar	O	2019	≤ 0.5	-	-	-	-	-	-	-	-	-
S389	*enterica*	Toulon	Wild Boar	S	2019	1	-	-	-	-	-	-	-	-	-
S390	*enterica*	Kottbus	Wild Boar	S	2019	2	-	-	-	-	-	-	-	-	-
S391	*houtenae (IV)*	1,40:z_4,z_{23}:-	Wild Boar	S	2020	2	-	-	-	-	-	-	-	-	-
S392	*houtenae (IV)*	1,40:z_4,z_{23}:-	Wild Boar	S	2020	≤ 0.5	-	-	-	-	-	-	-	-	-
S394	*houtenae (IV)*	1,40:z_4,z_{23}:-	Wild Boar	O	2020	≤ 0.5	-	-	-	-	-	-	-	-	-
S395	*enterica*	Infantis	Dog	S	2020	≤ 0.5	-	-	-	-	-	-	-	-	-
R12	*enterica*	Ceyco	Reptile	S	2001	1	-	-	-	-	-	-	-	-	-
R17	*enterica*	Muenchen	Reptile	S	2001	1	-	-	-	-	-	-	-	-	-
R25	*enterica*	Kapemba	Reptile	S	2001	≤ 0.5	-	-	-	-	-	-	-	-	-
R30	*enterica*	Friedrichsfelde	Reptile	S	2001	2	-	-	-	-	-	-	-	-	-
R32	*enterica*	Midway	Reptile	S	2001	2	-	-	-	-	-	-	-	-	-
R39	*enterica*	Trimndon	Reptile	S	2002	1	-	-	-	-	-	-	-	-	-
R40	*salamae (II)*	9,46:z_{39}:1,7	Reptile	S	2002	2	-	-	-	-	-	-	-	-	-
R43	*enterica*	Trimndon	Reptile	S	2002	≤ 0.5	-	-	-	-	-	+	-	-	-
R45	*enterica*	Caracas	Reptile	S	2002	2	-	-	-	-	-	-	-	-	-
R63	*enterica*	Berta	Reptile	S	2002	≤ 0.5	-	-	-	-	-	-	-	-	-
R90	*enterica*	Berta	Reptile	S	2002	≤ 0.5	-	-	-	-	-	-	-	-	-
R91	*enterica*	Berta	Reptile	S	2002	≤ 0.5	-	-	-	-	-	-	-	-	-
R92	*enterica*	Berta	Reptile	S	2002	≤ 0.5	-	-	-	-	-	-	-	-	-
R108	*enterica*	Memphis	Reptile	S	2002	≤ 0.5	-	+	-	-	-	-	-	-	-

R120	*enterica*	Senftenberg	Reptile	S	2002	≤ 0.5	-	-	-	-	-	-	-	-	-
R164	*houtenae (IV)*	44:z_4,z_{23}:-	Reptile	S	2002	2	-	-	-	-	-	-	-	+	-
R161	*houtenae (IV)*	44:z_4,z_{23}:-	Reptile	S	2002	2	-	+	-	-	-	-	-	-	-
R154	*houtenae (IV)*	44:z_4,z_{23}:-	Reptile	S	2002	256	-	-	-	-	-	-	-	-	-
R152	*enterica*	Berta	Reptile	S	2002	≤ 0.5	-	-	-	-	-	-	-	-	-
R133	*enterica*	Montevideo	Reptile	S	2002	≤ 0.5	-	-	-	-	-	-	-	-	-
R138	*enterica*	Kottbus	Reptile	S	2002	1	-	-	-	-	-	-	-	-	-
R144	*enterica*	Berta	Reptile	S	2002	≤ 0.5	-	-	-	-	-	-	-	-	-
R148	*enterica*	Berta	Reptile	S	2002	≤ 0.5	-	-	-	-	-	-	-	-	-
R140	*enterica*	Montevideo	Reptile	S	2002	1	-	-	-	-	-	-	-	-	-
R139	*enterica*	Chichiri	Reptile	S	2002	≤ 0.5	-	-	-	-	-	-	-	-	-
R173	*enterica*	Senftenberg	Reptile	S	2002	≤ 0.5	-	-	-	-	-	-	-	+	-
R121	*houtenae (IV)*	44:z_4,z_{23}:-	Reptile	S	2002	2	-	-	-	-	-	-	-	-	-
R174	*enterica*	Israel	Reptile	S	2002	2	-	-	-	-	-	-	-	-	-
R177	*enterica*	Anatum	Reptile	S	2002	1	-	-	-	-	-	-	-	-	-
R183	*enterica*	Kuntair	Reptile	S	2002	1	-	-	-	-	-	-	-	-	-
R193	*enterica*	Durban	Reptile	S	2002	2	-	-	-	-	-	-	-	-	-
R198	*enterica*	Berta	Reptile	S	2002	≤ 0.5	-	-	-	-	-	-	-	-	-
R199	*enterica*	Berta	Reptile	S	2002	≤ 0.5	-	-	-	-	-	-	-	-	-
R213	*enterica*	Agona	Reptile	S	2002	4	-	-	-	-	-	-	-	-	-
R221	*diarizonae (IIIb)*	50:r:e,n,x,z_{15}	Reptile	S	2002	≤ 0.5	-	-	-	-	-	-	-	-	-
R222	*houtenae (IV)*	44:z_4,z_{23}:-	Reptile	S	2002	2	-	-	-	-	-	-	-	-	-
R226	*houtenae (IV)*	44:z_4,z_{23}:-	Reptile	S	2002	2	-	-	-	-	-	-	-	-	-
R245	*enterica*	Othmarschen	Reptile	S	2002	2	-	-	-	-	-	-	-	-	-
R248	*enterica*	Othmarschen	Reptile	S	2002	1	-	-	-	-	-	-	-	-	-
R261	*enterica*	Midway	Reptile	S	2002	4	-	-	-	-	-	-	-	-	-
R263	*enterica*	Reading	Reptile	S	2002	1	-	-	-	-	-	-	-	-	-
R294	*enterica*	Caracas	Reptile	S	2002	1	-	-	-	-	-	-	-	-	-
R300	*diarizonae (IIIb)*	50:z:z_{52}	Reptile	S	2002	1	+	-	-	+	-	-	-	-	-
R278	*enterica*	Veneziana	Reptile	S	2002	2	-	-	-	-	-	-	-	-	-
R102	*salamae (II)*	48:d:z_6	Reptile	S	2002	2	-	-	-	-	-	-	-	-	-
R123	*houtenae (IV)*	44:z_4,z_{23}:-	Reptile	S	2002	1	-	-	-	-	-	-	-	-	-
R112	*houtenae (IV)*	18:z_{36},z_{23}:-	Reptile	S	2002	≤ 0.5	-	+	-	-	-	-	-	-	-

R86	*enterica*	Blukwa	Reptile	S	2002	≤ 0.5	-	-	-	-	-	-	-	-	-
R126	*diarizonae (IIIb)*	48:z_4,z_{23}:-	Reptile	S	2002	2	-	+	-	-	-	-	-	-	-
R52	*enterica*	Apapa	Reptile	S	2002	≤ 0.5	-	-	-	-	-	-	-	-	-
R99	*enterica*	Apapa	Reptile	S	2002	≤ 0.5	-	-	-	-	-	-	-	-	-
R103	*enterica*	Apapa	Reptile	S	2002	≤ 0.5	-	-	-	-	-	-	-	-	-
R296	*diarizonae (IIIb)*	50:b:z_6	Reptile	S	2002	≤ 0.5	-	-	-	-	-	-	-	-	-
R114	*salamae (II)*	16:g,m,s,t:-	Reptile	S	2002	≤ 0.5	-	-	-	-	-	-	-	-	-
R274	*enterica*	Fluntern	Reptile	S	2002	≤ 0.5	-	-	-	-	-	-	-	-	-
R279	*enterica*	Fluntern	Reptile	S	2002	1	-	-	-	-	-	-	-	-	-
R291	*enterica*	Ebrie	Reptile	S	2002	1	-	-	-	-	-	-	-	-	-
R142	*enterica*	Duval	Reptile	S	2002	1	-	-	-	-	-	-	-	-	-
R237	*enterica*	Ebrie	Reptile	S	2002	≤ 0.5	-	-	-	-	-	-	-	-	-
R210	*enterica*	Ebrie	Reptile	S	2002	2	-	-	-	-	-	-	-	-	-
R282	*salamae (II)*	50:b:z_6	Reptile	S	2002	≤ 0.5	-	-	-	-	-	-	-	-	-
R299	*salamae (II)*	50:b:z_6	Reptile	S	2002	2	-	-	-	-	-	-	-	-	-
R182	*enterica*	Fluntern	Reptile	S	2002	≤ 0.5	-	-	-	-	-	-	-	-	-
R137	*enterica*	Trimndon	Reptile	S	2002	1	-	-	-	-	-	-	-	-	-
S257	*enterica*	Typhimurium	Pigeon	O	2014	≤ 0.5	-	-	-	-	-	-	-	-	-
S347	*enterica*	London	Wild Boar	S	2018	≤ 0.5	-	-	-	-	-	-	-	-	-

Legend: TMV = Typhimurium monophasic variant; † = sick animal E = Environment, F = feed, O = Organs, O* = Entire subject, S = Faeces.
